# EAP and ECPCP Statement Risks for Children's Health During the COVID-19 Pandemic and a Call for Maintenance of Essential Pediatric Services

**DOI:** 10.3389/fped.2021.679803

**Published:** 2021-05-11

**Authors:** Łukasz Dembiński, Gottfried Huss, Igor Radziewicz-Winnicki, Zachi Grossman, Artur Mazur, Stefano del Torso, Shimon Barak, Angel Carrasco Sanz, Adamos Hadjipanayis

**Affiliations:** ^1^The European Academy of Paediatrics, EAP, Brussels, Belgium; ^2^Department of Pediatric Gastroenterology and Nutrition, Medical University of Warsaw, Warsaw, Poland; ^3^The European Confederation of Primary Care Paediatricians (ECPCP), Lyon, France; ^4^Kinder-Permanence Spital Zollikerberg, Zollikerberg, Switzerland; ^5^Medical Faculty, University of Rzeszow, Rzeszów, Poland; ^6^Adelson School of Medicine, Ariel University, Ariel, Israel; ^7^Maccabi Health Services, Tel Aviv, Israel; ^8^ChildCare WorldWide, Padova, Italy; ^9^Dana-Dwek Children's Hospital, Tel Aviv Sourasky Medical Center, Tel Aviv, Israel; ^10^Madrid Health Service, Servicio Madrileño de Salud, Madrid, Spain; ^11^School of Medicine, European University Cyprus, Nicosia, Cyprus; ^12^Paediatric Department, Larnaca General Hospital, Larnaca, Cyprus

**Keywords:** adolescents, community pediatrics, lockdown, mental health, pediatric primary care, SARS-CoV-2, school, vaccines

## Abstract

The COVID-19 pandemic and global lockdown have had drastic socioeconomic and psychological effects on countries and people, respectively. There has been limited access to health care and education. These negative consequences have had a significant impact on the well-being of children and adolescents. Therefore, the EAP and the ECPCP are requesting state, health, and education authorities as well as European pediatric societies and the healthcare professionals that special attention be given to this population and the problems they face as a result of the pandemic.

## Introduction

The COVID-19 pandemic has caused unprecedented changes in people's way of life and social relationships. Global public health measures, including a worldwide lockdown, have affected socioeconomics, health care, education, and public mental health (1–5). This has had a major impact on children and adolescents, affecting their physical, intellectual, and emotional development (6). Approximately 90% of children and adolescents, estimated as 1.5 billion young people worldwide, have been obliged to stay at home, significantly influencing their health and social functioning (7).

To highlight this issue, provide guidance, and offer strategies for the prevention or minimization of the detrimental effects of the pandemic and associated lockdown on young people, the European Academy of Paediatrics (EAP) and the European Confederation of Primary Care Paediatricians (ECPCP) present this position paper and recommendations directed to state, health authorities, European pediatric societies, and the European healthcare professionals involved in the care of the pediatric population. This statement does not focus on the care of children and adolescents with COVID-19 disease.

## Essential Pediatric CARE

The course of SARS-CoV-2 infection is rarely severe in children and adolescents (8–10). However, the long-term consequences of the limited access to essential medical care resulting from the pandemic restrictions should not be underestimated (11–16). Pediatric consultations in primary care and in hospitals dropped dramatically due to the fearfulness of parents and restrictions in the health care delivery (17). Primary medical care has largely been limited to remote consultations. This has resulted in delays to child presentations at emergency departments and specialist centers (18). The diagnosis of conditions such as diabetes mellitus, dehydration, appendicitis, sepsis, and neoplastic diseases have been significantly delayed in many instances (19–22). Limited access to health care has a particularly negative impact on newborns and young children (23, 24).

## Well-Child Care and Vaccination

The lockdown has also affected the availability of well-child care and the administration of childhood inoculations for vaccine-preventable diseases. Many children, especially those from lower-income households, have not received scheduled routine vaccinations and the overall immunization rate has dropped significantly (25–27). This puts children at risk for the resurgence of life-threatening vaccine-preventable diseases.

## Mental Health and Development in the Community

Limited access to mental health services for children and adolescents has resulted in the intensification of preexisting problems and an increase in the prevalence of depression and anxiety disorders (28–30).

The new situation has led to weakened communication, deprivation of personal peer relations, lack of physical activity, and reduced sensory stimulation. The consequences of this can include psychological deterioration, anxiety, frustration, stress disorder, adjustment disorders, grief, loss of appetite, and sleep problems (5, 31, 32). The adverse effects of social isolation are particularly great among adolescents as social relationships are extremely important during this stage of development (33, 34). These psychological and emotional difficulties are likely to be externalized as negative behavior. Many parents and caregivers are also experiencing greater stress because of the lockdown and home schooling and increased time with their children has caused many to feel uncertain of their parental abilities, which is especially apparent in the group characterized by the following factors: motherhood, being single, having younger children, having a special needs child, and having many children (35). These factors combined are liable to lead to intra-family conflicts and, in some cases, family violence (36–38). The data show an increase in the number of children exposed to violence and witnessing domestic violence during the pandemic. Whereas, limited school activities and reduced direct contact with primary healthcare may cause a decrease in child abuse victims' identification (39–41).

Social distancing and limited interpersonal contact can lead to development delays, impaired social skills, anxiety, and depressive symptoms (42). The lengthy, ongoing COVID-19 lockdown has caused a significant rise in suicidal ideation and behaviors among school children. Suicide and self-harm rates in minors have risen in both the general population and those with preexisting mental health disorders (43–45).

The transference of social activity exclusively to the Internet can result in digital technology overuse, abandonment of other activities, exposure to inappropriate content, and cyberbullying (46, 47).

## Safe Reopening of Schools During COVID-19 Pandemic

The prolonged school closures are likely to have detrimental effects on learning outcomes (48–50). In younger children, the presence of a teacher during educational activities is much more important. Children whose schooling has just begun require more time and attention from educators than older children (51).

The lockdown exacerbates socioeconomic inequalities. In the context of education, this can mean that children from poorer families are likely to have inadequate access to appropriate IT tools, insufficient intellectual support from caregivers, and fewer educational opportunities beyond school. As they do not receive meals at school during the lockdown, these children are also at increased risk of malnutrition (4, 52). Moreover, in children with special needs, such as autism spectrum disorders or learning disabilities, the lockdown-related transition to e-learning has been found to result in therapy discontinuation in many instances (32).

For teachers, the pandemic has been a challenge related to the crisis of competence. Educators have had to develop and improve objective methods of assessing learning progress. Remote education has also weakened teacher-student relationships (50, 53).

## Recommendations on Essential Pediatric Services

The EAP and the ECPCP strongly believe that action is necessary to reduce the negative effects of the pandemic on young people. The following measures are advised:

- For state, health, and education authorities

- Establish a framework on how to mitigate the impact of COVID-19 on children and adolescents needing essential services in a variety of healthcare settings.
- Consider strategies to reduce preventable illness during the COVID-19 pandemic in young people with non-COVID-19 diseases and injuries.
- Guarantee the staffing, adequate drug, and material supply, especially for personal protective equipment for essential pediatric services.
- Implement secure and flexible reopening and maintenance of kindergartens and schools based on the rights of children and adolescents.

- For European pediatric societies:

- Keep members updated in the area of risks for child and adolescent health during the COVID-19 pandemic.
- Encourage members to make all possible efforts to maintain curative and preventive high-quality services for children and adolescents.
- Initiate and coordinate European-wide surveys regarding the temporary impact of the COVID-19 pandemic on pediatric health services.
- Analyze the experiences with telemedicine in the area of pediatric care, screening, follow-up, and drug prescribing.
- Support the EAP campaign “Vaccinate your child,” to restore routine immunization of children (54).

- For the providers in the primary, secondary, and specialized pediatric care

- Increase vigilance and diligence about the risks of non-personal appointments when conducting remote health assessments of children and adolescents.
- Encourage face-to-face visits when presenting symptoms require them to rule out life-threatening diseases. Provide postnatal care in person wherever possible.
- Ensure hygienic measures and personal protection for all staff and patients. Make appropriate adjustments and appointments to avoid crowding in outpatient services. Limit the quantity of accompanying persons.
- Communicate personally and in public with caregivers to reassure the trust in essential health services and the unlikely danger of COVID-19 infection in the health care system. Clarify any public misinformation.
- Reinforce the capacity of parents and adolescents for self-management and timely presentation in case of alarming signs and symptoms.
- Make every effort to maintain essential pediatric health services by facilitating open access to curative services for the acute and the chronic ill children and adolescents.
- Maintain preventive health services as well as child checks and vaccinations. Use recall and other motivational reminder systems to catch-up with missed visits.
- Increase surveillance and screening for emotional, social, and behavioral disorders and provide age-appropriate guidance at any given opportunity.
- Engage to maintain mental health services and social services in the community, with special focus on lower-income households.
- Encourage adolescents to discuss non-pharmacological COVID-19 interventions such as social distancing, hygiene, and the use of face coverings. This will enable them to identify and minimize behavioral infection risks, which may be detrimental even in areas with high vaccination rates.

## Data Availability Statement

The original contributions presented in the study are included in the article/supplementary material, further inquiries can be directed to the corresponding author/s.

## Author Contributions

ŁD, AM, and AH: study design. ŁD, IR-W, and GH: data collection. ŁD, IR-W, GH, ZG, AM, ST, SB, AC, and AH: data analysis and interpretation, manuscript preparation, and critical revision. All authors read and approved the final manuscript.

## Conflict of Interest

The authors declare that the research was conducted in the absence of any commercial or financial relationships that could be construed as a potential conflict of interest.
